# Association between the severity of new-onset depression and unmet healthcare needs of South Korean adults

**DOI:** 10.1371/journal.pone.0256222

**Published:** 2021-08-19

**Authors:** Su Yeon Kim, Wonjeong Jeong, Eun-Cheol Park, Sohee Park, Sung-In Jang

**Affiliations:** 1 Health Insurance Review & Assessment Service, Gangwon, Republic of Korea; 2 Department of Public Health, Graduate School, Yonsei University, Seoul, Republic of Korea; 3 Institute of Health Services Research, Yonsei University, Seoul, Republic of Korea; 4 Department of Preventive Medicine, Yonsei University College of Medicine, Seoul, Republic of Korea; 5 Department of Biostatistics, Graduate School of Public Health, Yonsei University, Seoul, Republic of Korea; College of Medicine and Sagore Dutta Hospital, INDIA

## Abstract

**Objectives:**

Identifying whether the demand for medical services is catered to is an important issue. Given that depression is a major contributor to the overall global burden of disease, it could affect the use of healthcare. This study aims to examine the association between the severity of new-onset depression and unmet healthcare needs among South Korean adults.

**Methods:**

Data from 15,588 participants, derived from the 2014, 2016, and 2018 Korean National Health and Nutrition Examination Survey, were examined. Only individuals who were not diagnosed with depression was included to exclude those who visited hospitals to treat depression or were experiencing unmet healthcare needs due to depression. Depression was measured using the Patient Health Questionnaire-9 and unmet healthcare needs acted as the dependent variable. A multiple/multinomial logistic regression analysis was built to analyze the association between the variables.

**Results:**

Individuals with severe depression had a higher risk of having unmet healthcare needs compared to those without (men: adjusted OR = 2.05, 95% CI = 1.40–3.00; women: adjusted OR = 2.20, 95% CI = 1.72–2.82). White-collar men with severe depression also had a higher risk of having unmet healthcare needs (adjusted OR = 9.72, 95% CI = 4.73–20.00). Individuals with severe depression had a higher risk of having unmet healthcare needs due to economic hardship than those without depression (men: adjusted OR = 3.01, 95% CI = 1.76–5.14, women: adjusted OR = 2.93, 95% CI = 1.96–4.38).

**Conclusions:**

This study identified a significant relationship between the severity of new-onset depression and the risk of having unmet healthcare needs among South Korean adults. Our study suggests that having severe depression contributed to a higher risk of unmet healthcare needs. Proper care to manage depression can be promoted through future intervention programs that alleviate the risk of having unmet healthcare needs.

## Introduction

An unmet healthcare need is defined as the inability to receive medical services on time, despite medical needs or demand for medical services, which can lead to negative health consequences [1]. They are primarily due to the inaccessibility (e.g., cost, transportation) or unavailability (e.g., waiting time before receiving care) of services [1, 2]. Identifying whether the demand for medical services is met is of great importance at the national and individual levels [3].

In Korea, the medical insurance system was extended in 1989, allowing all citizens to have access to necessary medical services [1, 4]. While this has improved access to health care, problems of imbalance and low equity in the use of health care services continue to exist [3, 5]. The rate of Korea’s annual unmet healthcare needs has decreased; from 19.5% in 2007, it continued to decrease by an average of 9.1% every year, reaching 7.8% in 2018 [5]. However, as of 2017, the percentage of a household’s direct burden of current medical expenses is 33.7% in South Korea; this is much higher than 20.5%, which is the average according to the Organization for Economic Cooperation and Development (OECD) [6].

As modern society continually evolves, mental health, including depression and stress, has become a major social issue, especially in South Korea [7]. Depression is a leading cause of disability worldwide and a major contributor to the overall global burden of disease with more than 264 million people of all ages suffering from it [8]. Moreover, it can be associated with suicidal behaviors. Considering that South Korea recorded the second-highest suicide rate among OECD countries, it must focus on providing proper care for those who suffer from depression [9, 10].

Depression could affect economic burden due to its high prevalence and comorbidity with other conditions [11]. Moreover, its severity is highly associated with increased treatment cost, unemployment, and reduced performance at work, which could lead to unmet healthcare needs [12, 13]. Additionally, depression is highly associated with the underutilization of mental healthcare services, which contributes to it being a persistent concern. However, there are few studies on depression and unmet healthcare needs, excluding mental healthcare, using Korean data. For this reason, depression may have the greatest negative impact on time management and productivity [11].

Given this context, it is necessary to investigate the association between the severity of depression and unmet healthcare needs, which could prevent unmet healthcare needs, especially for those who have depression. This study hypothesized that having severe depression has a significant relation with unmet healthcare needs regardless of mental healthcare. Consequently, it sought to examine the association between the severity of depression and unmet healthcare needs in South Korean adults who have not been previously diagnosed with depression.

## Materials and methods

### Data and study participants

Data for this study were taken from the 2014, 2016, and 2018 Korean National Health and Nutrition Examination Survey (KNHANES). The KNHANES is a nationwide population-based survey conducted by the Korea Centers for Disease Control and Prevention (KCDC). It aims to evaluate the health and nutritional status of South Koreans, and provide data for the development and evaluation of health policies and programs in the country [14, 15]. Ethical approval was not required for this study because it used secondary data that are publicly available and de-identified.

The total number of participants surveyed in the 2014, 2016, and 2018 KNHANES was 23,692. As the Patient Health Questionnaire 9 (PHQ-9) was only investigated in 2014, 2016, and 2018, only 3 years were included in the analysis. We excluded information from individuals aged up to 18 years, and included data from participants aged 19 years or more. Moreover, we excluded data from respondents who were already diagnosed with depression to exclude those who visited hospitals to treat depression or were experiencing unmet healthcare needs due to depression. In addition, data with missing information on the following were also excluded: age, education level, marital status, number of household members, region, household income level, occupational status, health insurance type, private health insurance, smoking status, number of days of walking per week, number of days of muscle exercise, self-reported health status, stress level, and number of chronic diseases. Finally, data on a total of 15,558 participants (6,824 men and 8,734 women) were analyzed.

### Variables

The main independent variable of this study was depression, which was measured using the PHQ-9. The PHQ-9 is a nine-item self-administered version of the PHQ and has been validated as a reliable screening tool for depression, and measure of its severity [16]. Each item on the PHQ-9 is scored on a scale of 0 to 3, and the total severity score ranges from 0 to 27. Depression severity was defined as: no depression (0–4), mild depression (5–9), and severe depression (≥10) [17].

Additionally, the analyses included demographic, socioeconomic, and health-related characteristics. The demographic analysis considered age, sex, and number of household members. The socioeconomic analysis considered education level, marital status, region, household income level, occupation classification, type of health insurance, and private health insurance. Occupation classification was defined as white collar (manager, professionals, and office worker), blue collar (craft/trade workers, machine operators, and assemblers), and pink collar (services and sales workers), and none based on the International Standard Classification of Occupations [15, 18]. The health-related characteristics considered smoking status, number of days of walking per week, number of days of muscle exercise, self-reported health status, stress level and number of chronic diseases.

The dependent variable of this study was unmet healthcare needs. Individuals who answered “yes” to the questions, “During the past year, was there ever a time when you felt that you needed medical healthcare but did not receive it?” or “During the past year, was there ever a time when you felt that you needed dental healthcare but did not receive it?” were classified under the group who has experienced having unmet healthcare needs. Additionally, through the above questions, the types of unmet healthcare need were divided into only medical, only dental, and none. Respondents who have experienced unmet healthcare needs were asked to answer a follow-up question: “Thinking back to your experience of having unmet healthcare needs, why were you not able to receive healthcare?” Accordingly, the reasons for unmet healthcare needs were divided into economic hardship, lack of time, mild illness, other reason, and none.

### Statistical analysis

A chi-square test was conducted to investigate the general characteristics of the study population. A multiple logistic regression analysis was performed to examine the association of depression and unmet healthcare needs, after accounting for potential confounding variables including demographic, socioeconomic, and health-related characteristics. The factors associated with the reason for having unmet healthcare needs and type of unmet healthcare needs were analyzed using a multinomial logistic regression analysis. Multinomial logistic regressions were used when the dependent variables contained more than two categories. The results are reported as an odds ratio (OR) with a 95% confidence interval (CI). The analysis used a stratified sampling variable (kstrata) and clustering variable (primary sampling units) provided by the KNHANES. All analyses included the use of weighted variables. Differences were considered statistically significant with a p-value of <0.05. All data analyses were conducted using the SAS 9.4 software (version 9.4; SAS Institute Inc., Cary, NC, USA).

## Results

Table 1 presents the general characteristics of the study population according to sex. Among the participants (6,824 men and 8,734 women), 1,912 men (28.0%) and 3,008 women (34.4%) had unmet healthcare needs. The association between the severity of depression and unmet healthcare needs was statistically significant. In addition, the results were generally significant for the demographic, socioeconomic, and health-related characteristics.

**Table 1:** General characteristics of the study population.

Variables		Total (N = 15,558)		Men (N = 6,824)					Women (N = 8,734)				
				Unmet need				P-value	Unmet need				P-value
				Yes		No			Yes		No		
		N	(%)	N	(%)	N	(%)		N	(%)	N	(%)	
**Depression**			<0.0001					<0.0001					<0.0001
	No	12,761	(82.0)	1,521	(25.7)	4,391	(74.3)		2,071	(30.2)	4,778	(69.8)	
	Mild depression	2,065	(13.3)	276	(39.9)	416	(60.1)		637	(46.4)	736	(53.6)	
	Severe depression	732	(4.7)	115	(52.3)	105	(47.7)		300	(58.6)	212	(41.4)	
**Age (years)**			0.0005					0.0639					0.0008
	19–29	1,867	(12.0)	186	(22.8)	631	(77.2)		326	(31.0)	724	(69.0)	
	30–39	2,660	(17.1)	337	(28.9)	828	(71.1)		535	(35.8)	960	(64.2)	
	40–49	2,871	(18.5)	398	(32.0)	845	(68.0)		534	(32.8)	1,094	(67.2)	
	50–59	2,946	(18.9)	386	(31.2)	850	(68.8)		583	(34.1)	1,127	(65.9)	
	≥60	5,214	(33.5)	605	(25.6)	1,758	(74.4)		1,030	(36.1)	1,821	(63.9)	
**Educational level**			<0.0001					0.0123					<0.0001
	Middle school or less	4,416	(28.4)	468	(30.8)	1,052	(69.2)		1,129	(39.0)	1,767	(61.0)	
	High school	4,220	(27.1)	591	(31.3)	1,297	(68.7)		793	(34.0)	1,539	(66.0)	
	College or over	6,922	(44.5)	853	(25.0)	2,563	(75.0)		1,086	(31.0)	2,420	(69.0)	
**Marital status**			<0.0001					0.0008					<0.0001
	Married	11,052	(71.0)	1,409	(27.6)	3,693	(72.4)		2,011	(33.8)	3,939	(66.2)	
	Separated or divorced	1,969	(12.7)	140	(35.3)	257	(64.7)		629	(40.0)	943	(60.0)	
	Unmarried	2,537	(16.3)	363	(27.4)	962	(72.6)		368	(30.4)	844	(69.6)	
**Number of Household member**			<0.0001					<0.0001					<0.0001
	One person	1,681	(10.8)	215	(34.0)	418	(66.0)		397	(37.9)	651	(62.1)	
	Two person	3,569	(22.9)	466	(25.9)	1,332	(74.1)		579	(32.7)	1,192	(67.3)	
	Parents with Child	6,769	(43.5)	869	(27.5)	2,287	(72.5)		1,169	(32.4)	2,444	(67.6)	
	Others	3,539	(22.7)	362	(29.3)	875	(70.7)		863	(37.5)	1,439	(62.5)	
**Region**			0.1957					0.7770					0.4973
	Urban area	7,113	(45.7)	846	(27.3)	2,250	(72.7)		1,366	(34.0)	2,651	(66.0)	
	Rural area	8,445	(54.3)	1,066	(28.6)	2,662	(71.4)		1,642	(34.8)	3,075	(65.2)	
**Household income level**			<0.0001					<0.0001					<0.0001
	Low	2,730	(17.5)	321	(29.6)	763	(70.4)		670	(40.7)	976	(59.3)	
	Middle	8,257	(53.1)	1,104	(30.1)	2,560	(69.9)		1,626	(35.4)	2,967	(64.6)	
	High	4,571	(29.4)	487	(23.5)	1,589	(76.5)		712	(28.5)	1,783	(71.5)	
**Occupational classification**			<0.0001					0.0044					0.0121
	White-collar	3,829	(24.6)	514	(25.8)	1,476	(74.2)		585	(31.8)	1,254	(68.2)	
	Blue-collar	3,615	(23.2)	748	(32.6)	1,545	(67.4)		542	(41.0)	780	(59.0)	
	Pink-collar	2,094	(13.5)	203	(28.0)	522	(72.0)		496	(36.2)	873	(63.8)	
	None	6,020	(38.7)	447	(24.6)	1,369	(75.4)		1,385	(32.9)	2,819	(67.1)	
**Health Insurance type**			<0.0001					0.0003					<0.0001
	Health Insurance	15,102	(97.1)	1,859	(27.9)	4,794	(72.1)		2,869	(34.0)	5,580	(66.0)	
	Medical aid	456	(2.9)	53	(31.0)	118	(69.0)		139	(48.8)	146	(51.2)	
**Private Health Insurance**			0.0070					0.1806					<0.0001
	Yes	12,111	(77.8)	1,443	(27.7)	3,766	(72.3)		2,322	(33.6)	4,580	(66.4)	
	No	3,447	(22.2)	469	(29.0)	1,146	(71.0)		686	(37.4)	1,146	(62.6)	
**Smoking Status**			<0.0001					<0.0001					<0.0001
	Smoker	2,895	(18.6)	838	(34.1)	1,619	(65.9)		197	(45.0)	241	(55.0)	
	Ex-smoker	3,289	(21.1)	710	(25.2)	2,105	(74.8)		193	(40.7)	281	(59.3)	
	Never	9,374	(60.3)	364	(23.5)	1,188	(76.5)		2,618	(33.5)	5,204	(66.5)	
**Number of days of walking per week**			<0.0001					0.0173					<0.0001
	0–1	3,936	(25.3)	575	(32.9)	1,175	(67.1)		858	(39.2)	1,328	(60.8)	
	2–4	4,554	(29.3)	528	(27.7)	1,378	(72.3)		865	(32.7)	1,783	(67.3)	
	≥5	7,068	(45.4)	809	(25.5)	2,359	(74.5)		1,285	(32.9)	2,615	(67.1)	
**Number of days of muscle exercise**			<0.0001					0.0066					0.0147
	No	11,946	(76.8)	1,381	(29.9)	3,242	(70.1)		2,612	(35.7)	4,711	(64.3)	
	1–3	2,086	(13.4)	287	(24.6)	879	(75.4)		264	(28.7)	656	(71.3)	
	≥4	1,526	(9.8)	244	(23.6)	791	(76.4)		132	(26.9)	359	(73.1)	
**Self-Reported Health Status**			<0.0001					<0.0001					<0.0001
	High	4,674	(30.0)	493	(22.0)	1,752	(78.0)		628	(25.9)	1,801	(74.1)	
	Middle	8,097	(52.0)	987	(28.2)	2,511	(71.8)		1,573	(34.2)	3,026	(65.8)	
	Low	2,787	(17.9)	432	(40.0)	649	(60.0)		807	(47.3)	899	(52.7)	
**Stress Level**			<0.0001					<0.0001					<0.0001
	High	3,804	(24.5)	564	(36.9)	965	(63.1)		985	(43.3)	1,290	(56.7)	
	Middle	8,875	(57.0)	1,067	(26.9)	2,901	(73.1)		1,645	(33.5)	3,262	(66.5)	
	Low	2,879	(18.5)	281	(21.2)	1,046	(78.8)		378	(24.4)	1,174	(75.6)	
**Number of Chronic Disease**			0.0653					0.7448					0.0004
	0	8,748	(56.2)	1,098	(28.5)	2,754	(71.5)		1,639	(33.5)	3,257	(66.5)	
	1–2	5,329	(34.3)	680	(28.2)	1,728	(71.8)		995	(34.1)	1,926	(65.9)	
	≥ 3	1,481	(9.5)	134	(23.8)	430	(76.2)		374	(40.8)	543	(59.2)	
**Year**			<0.0001					0.1025					<0.0001
	2014	4,540	(29.2)	618	(31.9)	1,319	(68.1)		1,027	(39.5)	1,576	(60.5)	
	2016	5,405	(34.7)	650	(27.3)	1,730	(72.7)		971	(32.1)	2,054	(67.9)	
	2018	5,613	(36.1)	644	(25.7)	1,863	(74.3)		1,010	(32.5)	2,096	(67.5)	
**Total**		**15,558**	**(100.0)**	**1,912**	**(28.0)**	**4,912**	**(72.0)**		**3,008**	**(34.4)**	**5,726**	**(65.6)**	

Table 2 shows the association between the severity of depression and unmet healthcare needs. People who had severe depression had the highest risk of having unmet healthcare needs (men: adjusted OR = 2.05, 95% CI = 1.40–3.00; women: adjusted OR = 2.20, 95% CI = 1.72–2.82), followed by those who had mild depression (men: adjusted OR = 1.52, 95% CI = 1.24–1.85; women: adjusted OR = 1.68, 95% CI = 1.44–1.96). These results were significant for both sexes. Individuals with a low level of household income (men: adjusted OR = 1.30, 95% CI = 1.02–1.66; women: adjusted OR = 1.36, 95% CI = 1.11–1.67), and those who had a low self-reported health status (men: adjusted OR = 1.86, 95% CI = 1.49–2.32; women: adjusted OR = 1.86, 95% CI = 1.55–2.22) also had a higher risk of having unmet healthcare needs than those with a high income and self-reported health status, respectively.

**Table 2:** Factors associated with unmet healthcare needs.

Variables		Men				Women			
		Unmet need				Unmet need			
		Adjusted OR	95% CI			Adjusted OR	95% CI		
**Depression**									
	No	1.00				1.00			
	Mild depression	1.52	(1.24	–	1.85)	1.68	(1.44	–	1.96)
	Severe depression	2.05	(1.40	–	3.00)	2.20	(1.72	–	2.82)
**Age (years)**									
	19–29	0.90	(0.63	–	1.29)	1.05	(0.77	–	1.42)
	30–39	1.13	(0.87	–	1.48)	1.17	(0.92	–	1.49)
	40–49	1.38	(1.09	–	1.76)	1.05	(0.83	–	1.32)
	50–59	1.31	(1.06	–	1.61)	1.02	(0.85	–	1.23)
	≥60	1.00				1.00			
**Educational level**									
	Middle school or less	1.18	(0.95	–	1.48)	1.12	(0.92	–	1.38)
	High school	1.18	(0.99	–	1.41)	1.00	(0.86	–	1.16)
	College or over	1.00				1.00			
**Marital status**									
	Married	1.00				1.00			
	Separated or divorced	1.24	(0.88	–	1.76)	1.13	(0.93	–	1.39)
	Unmarried	1.07	(0.83	–	1.37)	0.75	(0.59	–	0.96)
**Number of Household member**									
	One person	1.11	(0.80	–	1.53)	1.10	(0.86	–	1.41)
	Two person	1.00				1.00			
	Parents with Child	0.95	(0.78	–	1.16)	1.19	(1.00	–	1.40)
	Others	0.95	(0.75	–	1.20)	1.28	(1.06	–	1.54)
**Region**									
	Urban area	1.00				1.00			
	Rural area	1.01	(0.89	–	1.14)	1.05	(0.94	–	1.17)
**Household income level**									
	Low	1.30	(1.02	–	1.66)	1.36	(1.11	–	1.67)
	Middle	1.29	(1.10	–	1.50)	1.24	(1.08	–	1.43)
	High	1.00				1.00			
**Occupational classification**									
	White-collar	1.13	(0.88	–	1.43)	1.14	(0.97	–	1.34)
	Blue-collar	1.29	(1.05	–	1.59)	1.53	(1.29	–	1.80)
	Pink-collar	1.08	(0.82	–	1.43)	1.27	(1.08	–	1.50)
	None	1.00				1.00			
**Health Insurance type**									
	Health Insurance	1.00				1.00			
	Medical aid	0.69	(0.42	–	1.13)	1.38	(1.04	–	1.84)
**Private Health Insurance**									
	Yes	1.00				1.00			
	No	1.22	(1.01	–	1.47)	0.99	(0.85	–	1.17)
**Smoking Status**									
	Smoker	1.27	(1.07	–	1.51)	1.30	(1.02	–	1.65)
	Ex-smoker	0.95	(0.79	–	1.15)	1.17	(0.91	–	1.50)
	Never	1.00				1.00			
**Number of days of walking per week**									
	0–1	1.00				1.00			
	2–4	0.88	(0.74	–	1.04)	0.89	(0.76	–	1.04)
	≥5	0.84	(0.72	–	0.98)	0.92	(0.80	–	1.06)
**Number of days of muscle exercise**									
	No	1.00				1.00			
	1–3	0.93	(0.78	–	1.11)	0.87	(0.72	–	1.04)
	≥4	0.92	(0.76	–	1.12)	0.75	(0.59	–	0.97)
**Self-Reported Health Status**									
	High	1.00				1.00			
	Middle	1.15	(0.98	–	1.33)	1.35	(1.18	–	1.54)
	Low	1.86	(1.49	–	2.32)	1.86	(1.55	–	2.22)
**Stress Level**									
	High	1.62	(1.29	–	2.02)	1.60	(1.32	–	1.93)
	Middle	1.28	(1.07	–	1.53)	1.48	(1.25	–	1.76)
	Low	1.00				1.00			
**Number of Chronic Disease**									
	0	1.60	(1.20	–	2.14)	1.10	(0.89	–	1.35)
	1–2	1.56	(1.18	–	2.07)	0.92	(0.77	–	1.11)
	≥3	1.00				1.00			
**Year**									
	2014	1.36	(1.16	–	1.59)	1.42	(1.24	–	1.63)
	2016	0.95	(0.81	–	1.10)	0.98	(0.86	–	1.11)
	2018	1.00				1.00			

Table 3 shows the results of the subgroup analyses for the severity of depression and unmet healthcare needs stratified by household income level, occupational classification, number of days of muscle exercise, and stress level. Respondents of both sexes with low income who had severe depression had a higher risk of having unmet healthcare needs (men: adjusted OR = 3.49, 95% CI = 1.58–7.71; women: adjusted OR = 3.28, 95% CI = 1.98–5.45). White-collar male workers with severe depression (adjusted OR = 9.72, 95% CI = 4.73–20.00), and severely depressed individuals with a high stress level (men: adjusted OR = 5.33, 95% CI = 3.31–8.59; women: adjusted OR = 2.70, 95% CI = 1.82–4.00) were also more at risk of having unmet healthcare needs, compared to those without depression.

**Table 3:** Subgroup analysis associations between depression and unmet healthcare needs stratified by covariates.

Variables		No	Mild depression				Severe depression			
			Adjusted OR	95% CI			Adjusted OR	95% CI		
**Men**										
**Household income level**										
	Low	1.00	1.90	(0.99	–	3.63)	3.49	(1.58	–	7.71)
	Middle	1.00	2.36	(1.59	–	3.51)	4.53	(2.62	–	7.84)
	High	1.00	0.75	(0.37	–	1.54)	3.97	(1.42	–	11.13)
**Occupational classification**										
	White-collar	1.00	1.54	(0.92	–	2.59)	9.72	(4.73	–	20.00)
	Blue-collar	1.00	1.51	(0.85	–	2.68)	3.53	(1.60	–	7.77)
	Pink-collar	1.00	3.14	(1.35	–	7.30)	4.63	(1.23	–	17.46)
	None	1.00	2.16	(1.17	–	3.97)	3.21	(1.50	–	6.88)
**Number of days of muscle exercise**										
	No	1.00	1.86	(1.29	–	2.67)	4.29	(2.72	–	6.78)
	1–3	1.00	2.20	(1.01	–	4.79)	2.74	(0.72	–	10.39)
	≥4	1.00	1.26	(0.48	–	3.33)	4.32	(1.14	–	16.44)
**Stress Level**										
	High	1.00	1.97	(1.34	–	2.92)	5.33	(3.31	–	8.59)
	Middle	1.00	1.68	(1.02	–	2.79)	3.05	(1.31	–	7.14)
	Low	1.00	1.73	(0.32	–	9.46)	3.56	(0.36	–	34.91)
**Women**										
**Household income level**										
	Low	1.00	1.73	(1.08	–	2.78)	3.28	(1.98	–	5.45)
	Middle	1.00	1.92	(1.45	–	2.55)	3.01	(2.02	–	4.47)
	High	1.00	2.05	(1.28	–	3.30)	1.71	(0.73	–	3.99)
**Occupational classification**										
	White-collar	1.00	2.05	(1.21	–	3.48)	2.87	(1.30	–	6.34)
	Blue-collar	1.00	2.98	(1.95	–	4.54)	4.70	(2.27	–	9.74)
	Pink-collar	1.00	1.90	(1.14	–	3.18)	4.29	(2.17	–	8.46)
	None	1.00	1.57	(1.14	–	2.17)	2.14	(1.42	–	3.21)
**Number of days of muscle exercise**										
	No	1.00	2.06	(1.62	–	2.63)	2.65	(1.95	–	3.60)
	1–3	1.00	1.51	(0.70	–	3.22)	3.47	(1.26	–	9.58)
	≥4	1.00	0.70	(0.16	–	3.05)	1.95	(0.26	–	14.65)
**Stress Level**										
	High	1.00	1.90	(1.32	–	2.72)	2.70	(1.82	–	4.00)
	Middle	1.00	2.07	(1.51	–	2.84)	3.18	(1.76	–	5.74)
	Low	1.00	1.69	(0.67	–	4.23)	1.90	(0.63	–	5.74)

Table 4 shows the association between the severity of depression and reasons for having unmet healthcare needs according to sex. People with severe depression had a higher risk of having unmet healthcare needs due to economic hardship (men: adjusted OR = 3.01, 95% CI = 1.76–5.14; women: adjusted OR = 2.93, 95% CI = 1.96–4.38). Furthermore, those who had severe depression had a higher risk of having unmet healthcare needs due to lack of time as compared to those without depression (men: adjusted OR = 1.72, 95% CI = 0.96–3.09; women: adjusted OR = 2.48, 95% CI = 1.70–3.61).

**Table 4:** Factors associated with the reason for unmet healthcare needs^a^.

Variables		Economic hardship				Lack of time				Mild illness				Other reason			
		Adjusted OR	95% CI			Adjusted OR	95% CI			Adjusted OR	95% CI			Adjusted OR	95% CI		
Men																	
**Depression**																	
	No	1.00				1.00				1.00				1.00			
	Mild depression	1.69	(1.16	–	2.47)	1.68	(1.25	–	2.26)	1.41	(0.96	–	2.08)	1.32	(0.86	–	2.02)
	Severe depression	3.01	(1.76	–	5.14)	1.72	(0.96	–	3.09)	1.42	(0.69	–	2.93)	1.13	(0.50	–	2.58)
**Women**																	
**Depression**																	
	No	1.00				1.00				1.00				1.00			
	Mild depression	1.98	(1.53	–	2.57)	1.77	(1.38	–	2.28)	1.55	(1.16	–	2.06)	1.22	(0.92	–	1.62)
	Severe depression	2.93	(1.96	–	4.38)	2.48	(1.70	–	3.61)	1.76	(1.10	–	2.83)	1.67	(1.11	–	2.51)

^a^Reason for unmet healthcare need is analyzed by multinomial logistic regression. The reference is "no" unmet healthcare need.

Table 5 shows the association between the severity of depression and type of unmet healthcare need according to sex. Those who had severe depression had a higher risk of experiencing both medical and dental unmet healthcare needs (men: adjusted OR = 5.95, 95% CI = 3.71–9.55; women: adjusted OR = 4.91, 95% CI = 3.36–7.19).

**Table 5:** Factors associated with the type of unmet healthcare needs^a^.

Variables		Both				Only Medical				Only Dental			
		Adjusted OR	95% CI			Adjusted OR	95% CI			Adjusted OR	95% CI		
Men													
**Depression**													
	No	1.00				1.00				1.00			
	Mild depression	2.02	(1.40	–	2.93)	1.86	(1.15	–	3.00)	1.38	(1.09	–	1.74)
	Severe depression	5.95	(3.71	–	9.55)	1.53	(0.61	–	3.81)	1.27	(0.79	–	2.04)
**Women**													
**Depression**													
	No	1.00				1.00				1.00			
	Mild depression	2.73	(2.02	–	3.69)	1.67	(1.23	–	2.28)	1.48	(1.26	–	1.74)
	Severe depression	4.91	(3.36	–	7.19)	2.02	(1.29	–	3.16)	1.63	(1.22	–	2.18)

^a^ Type of unmet healthcare need is analyzed by multinomial logistic regression. The reference is "no" unmet healthcare need.

## Discussion

Depression is a major contributor to the overall global burden of disease, which could affect the economy [8]. As the primary reason for having unmet healthcare needs is economic hardship, it is necessary to identify the relationship between depression and unmet healthcare needs [1, 2]. Therefore, the aim of this study was to show this association among South Korean adults who have not been diagnosed with depression. Subsequently, we analyzed the association between the severity of depression and type of unmet healthcare need, which could provide detailed evidence to support those who experience having unmet healthcare needs.

While healthcare should be delivered in an acceptable, available, and cost-effective manner to everyone, people worldwide still have unmet needs, which is defined as being unable to obtain care when people believed it to be medically necessary [19]. Our study found that white-collar men were more at risk of having unmet healthcare needs when they had severe depression. Previous study shows that Korean white-collar workers tend to have high depression and low self-efficiency due to the high level of stress caused by simplified and repetitive work and lack of autonomy [20]. Moreover, especially among white-collar workers, as the responsibilities associated with jobs have increased, the employees tend to have longer working hours [21]. Long working hours is an emerging issue in Korea, and awareness on the importance of work and life balance has increased [21]. However, compared to the OECD statistics (1,734 hours), the average number of hours worked per year in Korea is still higher by 259 hours [22]. Therefore, although they need medical intervention—especially the ones with depressive symptoms—most workers lacked the time to visit hospitals and avail medical services.

The severity of depression is highly associated with economic hardships because depression is usually more common among those with low income [23]. Various mental illnesses such as anxiety, depression, fatigue, and burnout could affect working productivity, which could lead to economic problems [24]. Our study also found a significant association between people with severe depression and their experience of having unmet healthcare needs due to economic hardship. A previous study shows that the United States exhibited the largest inequality of medical use associated with income level, with a third of adults among the lower 50% of the population in terms of income level experiencing unmet medical needs due to financial reasons [19]. Experiencing economic problems, such as having unmet healthcare needs due to economic hardship, could more seriously affect depression, creating a vicious cycle [25]. Therefore, there is an urgent need to prevent those who suffer from depression from experiencing unmet healthcare needs.

Our study also found that those who had severe depression had a higher risk of having both medical and dental unmet healthcare needs. Likewise, depression is highly associated with unmet needs of both medical and dental care. While the importance of dental care is usually underestimated compared to medical care, mental illness often makes people fearful of visiting the dentist and undergoing dental treatment [26]. Moreover, a previous study showed that mental illness is associated with poor oral health because of a lack of access to dental care [26, 27].

It should be noted that the current study had several limitations. First, the results were based on self-reporting; thus, some survey questions might be subject to recall bias, especially for health-related characteristics. As these responses can also be affected by social desirability bias, caution should be taken when interpreting the results. Second, due to this study’s cross-sectional design, the cause, effect, and directionality of the relationships observed cannot be determined. Last, due to limited survey questions, it is difficult to distinguish whether having unmet healthcare needs can also mean a delay in medical care or not receiving any at all.

Despite these limitations, our study still has its strengths. The KNHANES is conducted by a national institution based on random cluster sampling; the data are statistically reliable and representative compared to surveys performed by private institutions [28]. Furthermore, data from the KNHANES were obtained from health interviews, which include physical examinations and nutrition surveys that can be used as a basis for developing health-related policies or programs [28, 29]. Moreover, as this study excluded those already diagnosed with depression, the results could show their unmet healthcare needs without the need to visit a hospital for psychiatric purposes. Therefore, our study could control the visiting hospital due to depression.

The current study identified a significant relationship between the severity of new-onset depression and the unmet healthcare needs of South Korean adults. Our findings suggest that people with severe depression are most at risk of having unmet healthcare needs compared to those without depression. Moreover, severe depression was highly associated with unmet healthcare needs due to economic hardship. Although the medical insurance system was extended to all citizens of Korea, unmet healthcare needs remains a major problem in the country. Our study suggests future interventions to alleviate the burden of unmet healthcare needs especially for those with depressive symptoms.
